# Endogenous Digitalis-Like Factors: An Overview of the History

**DOI:** 10.3389/fendo.2015.00049

**Published:** 2015-04-13

**Authors:** Vardaman M. Buckalew

**Affiliations:** ^1^Medical Center Boulevard, Wake Forest School of Medicine, Winston Salem, NC, USA

**Keywords:** natriuretic hormone, digitalis-like factor, ouabain, marinobufagenin, bufodienolides, cardenolides

## Abstract

The sodium pump is a ubiquitous cell surface enzyme, a Na, K ATPase, which maintains ion gradients between cells and the extracellular fluid (ECF). The extracellular domain of this enzyme contains a highly conserved binding site, a receptor for a plant derived family of compounds, the digitalis glycosides. These compounds inhibit the enzyme and are used in the treatment of congestive heart failure and certain cardiac arrhythmias. The highly conserved nature of this enzyme and its digitalis receptor led to early suggestions that endogenous regulators might exist. Recent examination of this hypothesis emerged from research in two separate areas: the regulation of ECF volume by a natriuretic hormone (NH), and the regulation of peripheral vascular resistance by a circulating inhibitor of vascular Na, K ATPase. These two areas merged with the hypothesis that NH and the vascular Na, K ATPase inhibitor were in fact the same entity, and that it played a causative role in the pathophysiology of certain types of hypertension. The possibility that multiple endogenous digitalis-like factors (EDLFs) exist emerged from efforts to characterize the circulating enzyme inhibitory activity. In this review, the development of this field from its beginnings is traced, the current status of the structure of EDLFs is briefly discussed, and areas for future development are suggested.

## Background

The regulation of salt and water excretion by the kidneys has occupied investigators since at least from the beginning of the 20th century. Highlights of these investigations are documented in early reviews of the subject, notably those by Epstein (1) and Smith (2) in the 1950s. These reviews supported the existence of a receptor-integrator-effector reflex by which changes in some component of the extracellular fluid (ECF) volume (“volume receptors”) caused appropriate changes in renal sodium excretion. Both “efferent factors,” the numerous hemodynamic, humoral, and neural factors known to directly affect renal sodium excretion, and “afferent factors,” the stimuli that activate the efferent factors, were reviewed.

Smith, separating the factors influencing free water excretion from those affecting sodium excretion, considered the mechanism of the latter as being similar to the former. Based on these and other evolutionary considerations, he postulated that the proposed effector for sodium excretion, which he called “Hormone X”, was an anti-natriuretic hormone, analogous to antidiuretic hormone, which had evolved to conserve sodium as our primitive ancestors made their “ascent through the brackish waters of the estuary/to the salt poor lakes and ponds” (Strauss) (2). Aldosterone had been identified in the early 1950s, so Smith’s Hormone X was clearly proposed as an additional volume sensitive sodium retaining hormone, decreased levels of which would cause natriuresis in response to increased ECF volume.

At the time of Smith’s review, it was well established that two factors were preeminent in controlling renal sodium excretion, glomerular filtration rate (GFR), and aldosterone. Thus, Smith’s review set the stage for exploration for a “third factor,” a term which did not originate with Smith. The earliest investigators to use the term in print, if not the first, were Bricker et al., who were searching for the mechanisms contributing to the progressive increase in the absolute rate of sodium excretion per nephron as the nephron population decreased in chronic renal failure (3). Bricker et al. were the first to recognize that similar mechanisms might contribute to both volume expansion natriuresis and the renal adaptation to chronic renal failure.

## The Concept of Natriuretic Hormone

Four years after Smith’s review, the mechanism of “volume expansion natriuresis” was addressed in a classic paper by deWardener et al. published in 1961 (4). They showed that natriuresis caused by saline infusion in dogs given large doses of mineralocorticoid was not abolished when GFR was reduced below initial levels by constriction of the aorta above the renal arteries. Furthermore, they showed that blood circulated from volume expanded dogs (donor) to euvolemic dogs (recipient) caused natriuresis in the recipient. Based on these studies, deWardener et al. suggested that volume expansion increased the circulating level of some natriuretic substance, and the concept of “natriuretic hormone” was born.

Three problems quickly emerged after this ground-breaking study was published. First, although the cross circulation studies were careful to control the volume of the recipient dog, the possible effects of blood dilution by the saline infusion in the donor dog were not. In addition, the possibility that the natriuresis in the recipient dog might be due to suppression of an anti-natriuretic factor as suggested by Smith had not been definitively eliminated. Each of these issues was addressed in the burst of work in other laboratories that followed the original paper by de Wardener et al. Importantly, these investigators effectively dealt with the dilution issue in a subsequent cross circulation study in which the recipient dog was infused with blood from a reservoir in which blood from both donor and recipient was in equilibrium (5). Other studies addressing the issue of dilution were published by Lichardus et al. and others (6). Studies of the effects of blood dilution on renal sodium excretion subsequently led to exploration of the so called “physical factors” on renal tubular sodium reabsorption by a number of laboratories (7).

In essentially all refinements of the cross circulation studies, the natriuresis in the recipient was much less than that in the donor animal, a finding that was never entirely explained, but some interesting observations were made. For example, response in the recipient was increased by infusing blood from the donor into the aorta just above the renal arteries (8), suggesting a short biologic half-life of the circulating natriuretic factor (9). Also, recipient response was enhanced by preventing the donor from excreting the administered volume load, suggesting some effect of “sustained” volume expansion, an interesting but poorly defined concept that has not been explored further (10).

### Mechanisms of natriuresis

The cross circulation studies did not distinguish between the presence of a natriuretic substance versus suppression of an anti-natriuretic substance. To make that distinction, a number of laboratories reported natriuretic activity in plasma, urine, and/or kidney tissue of volume expanded animals (11–13), the mechanism of which drew immediate interest. The question was whether the factor caused changes in renal hemodynamics or directly inhibited tubular sodium transport systems. The first studies suggesting the latter were performed by Bricker et al. in which inhibition of p-aminohippurate (PAH) transport by rabbit kidney cortical slices was inhibited by plasma from volume expanded subjects (14). Inhibition of transport in renal tubular epithelium was subsequently shown in isolated tubular cells (15).

Other early studies utilized anuran membranes as models of renal tubular sodium transport. Cort and Lichardus reported inhibition of sodium transport as measured by Ussing’s short circuit current (SCC) technique in isolated frog skin by deproteinized, concentrated plasma extracts with very high sodium concentrations (16). In more extensive studies using plasma ultrafiltrates with physiological salt concentrations from volume expanded dogs, Buckalew et al. in 1970 showed similar effects on toad bladder SCC of *bufo marinus* (17). Ussing and others had demonstrated that the SCC in anuran membrane was due to active sodium transport, and could be inhibited by ouabain. The demonstration that the putative natriuretic hormone inhibited tubular transport and SCC set the stage for investigation of the effect of this factor or factors on Na, K ATPase. The initial attempts to relate natriuretic hormone (NH) to Na, K ATPase inhibition were unsuccessful. In the best documented studies, Katz et al. were unable to show inhibition of the enzyme in renal cortical microsomes from volume expanded dogs and rats, or an effect of plasma dialyzates from these animals on renal microsomal Na, K ATPase isolated from euvolemic animals (18). However, Gonick et al. subsequently reported that a natriuretic fraction extracted from renal tissue and plasma of volume expanded animals inhibited SCC in frog skin and ouabain sensitive Na, K ATPase isolated from whole rat kidney (19, 20).

Studies of the effect of plasma, and extracts of plasma and urine of volume expanded subjects on sodium excretion in assay animals, usually rats, demonstrated two basic patterns that differed primarily in time to peak and duration of effect. The shorter acting pattern showed an immediate onset, a peak effect in 40–60 min, and duration of about 120 min (21, 22). The longer-acting pattern exhibited a delay in onset of 10–60 min, a peak effect in 2–3 h, and duration longer than 3 h (22). Some initial purification studies indicated that the more rapidly acting factor was found in fractions containing low molecular weight substances, and the longer acting factor appeared in fractions containing high molecular weight substances (23).

## Natriuretic Hormone as Inhibitor of Na, K ATPase

Two major developments in the late 1970s and early 1980s caused a shift in the direction of NH research. The discovery in 1981 of atrial natriuretic factor (ANF) by DeBold et al. (24) and its subsequent characterization as a peptide signaling cascade present in many organs displaced most other lines of investigation with regard to the existence and nature of a NH. Early studies did not show an effect of ANF on Na, K ATPase (25, 26); however, subsequent studies revealed a more complex situation (27–29). Nevertheless, it was clear from the early work that ANF and the natriuretic inhibitor of renal epithelial transport systems dependent on Na, K ATPase were two entirely different systems. However, very little further work on the non-ANF NH hypothesis was performed. Instead, the focus shifted to the second major development, namely, that NH might be an inhibitor of vascular Na, K ATPase that could also be a causative factor in certain types of hypertension.

The suggestion that some types of hypertension, especially those associated with ECF volume expansion, might be due to a circulating inhibitor of vascular Na, K ATPase evolved from studies of the phenomenon of potassium-induced vasodilation. Overbeck et al. showed that the dilator response to potassium, but not to other agents, was suppressed in the forelimb of the rat with two kidney, one clip hypertension and the dog with one-kidney, one-wrap hypertension (30). Subsequent studies showed that potassium-induced vasodilation was completely blocked by ouabain, leading to the hypothesis that the vasodilation was due to stimulation of vascular smooth muscle Na, K ATPase. According to this hypothesis, stimulation of the electrogenic sodium pump led to hyperpolarization, decreased voltage sensitive influx of calcium, and hence vascular relaxation (31).

**Figure 1:**
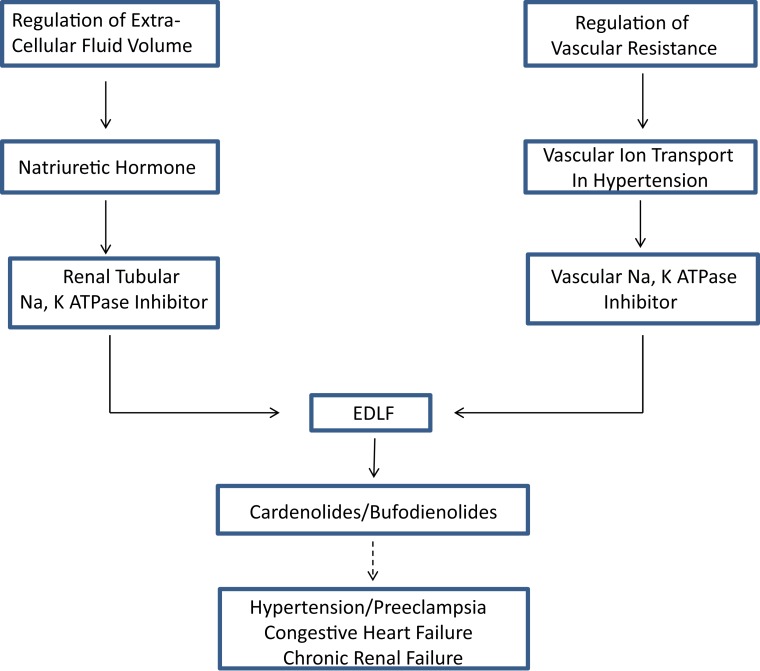
**The concept of an endogenous digitalis-like factor (EDLF) that inhibits Na, K ATPase in a manner similar to the cardiac glycosides developed from two lines of investigation (see text), the response of renal sodium excretion to extracellular fluid volume expansion, and the regulation of peripheral vascular resistance in hypertension**. Research on the identity of EDLF indicates that mammalian species synthesize two classes of steroids that are either identical to, or analogs of those found in plants (cardenolides) and toads (bufodienolides). Evidence suggests that one or more of these compounds may be involved in the pathophysiology of various hypertensive disorders, chronic renal failure, and congestive heart failure.

Reduced serum potassium produced identical effects in the opposite direction. That is, hypokalemia was associated with vasoconstriction and suppressed Na pump activity, suggesting a cause and effect relationship. As predicted by this paradigm, vascular depolarization was found in several volume expanded hypertension models. Thus, the hypothesis was proposed that vasoconstriction leading to hypertension might be caused by generalized inhibition of vascular Na, K ATPase activity (32). In a further refinement of the hypothesis in 1976, based on a review of then existing evidence for a humoral factor that slowly increased blood pressure in both animal models and humans with hypertension, Haddy et al. proposed that Na, K ATPase inhibition in vascular tissue, and hence vasoconstriction, might be due to a circulating factor (33). They, in fact, proposed in that review that the postulated circulating inhibitor of Na, K ATPase might be “natriuretic hormone.” Thus, the two fields of ECF volume regulation and regulation of vascular tone in volume expanded models of hypertension were brought together in the search for a common, explanatory factor (Figure 1).

A unifying explanation for the connection between vascular tone, intracellular calcium concentration, and the sodium pump was proposed by Blaustein in 1977 (34). The model was based on the presence of a Na–Ca exchanger located in the plasma membrane, driven by the intracellular–extracellular sodium gradient. According to the hypothesis, supported by kinetic calculations (34), inhibition of the sodium pump by the NH would cause increased vasoconstriction by inhibiting the outward transport of calcium by the Na–Ca exchanger.

### Volume expanded models of hypertension

The NH hypothesis of hypertension raised the question of how volume regulation by a potentially vasoconstrictor NH occurred in normal versus hypertensive subjects. Volume expanded models of hypertension involved some manipulation that reduced the ability of the kidney to excrete sodium. This approach was based on the concept proposed by Guyton et al. (35) that all hypertension was caused by an abnormal relationship between blood pressure and renal sodium excretion. According to this hypothesis, in normal subjects, renal adaptation to increases and decreases in sodium intake occur without any or with only small changes in systemic blood pressure. However, increased blood pressure is required to maintain ECF volume regulation in the presence of impaired renal sodium excretion through the phenomenon of “pressure diuresis.” Guyton postulated the rise in pressure was due to a volume induced increase in cardiac output and the consequent “long term autoregulation” (i.e., vasoconstriction) that ensued. Thus, ECF volume is maintained at the expense of increased peripheral vascular resistance and high blood pressure.

Based on this theory, several investigators proposed a unifying hypothesis incorporating NH that explained many observations then existing in the literature (36, 37). According to this formulation, the defect in renal response to increases in sodium and water intake in hypertensive subjects leads to increases in NH, vascular Na, K ATPase inhibition, vasoconstriction, and increased blood pressure. Volume homeostasis is maintained in the presence of a defect in renal sodium excretion by both the rise in blood pressure through the mechanism of pressure natriuresis, and by the effect of the NH to inhibit renal tubular sodium reabsorption. The difference between hypertensive and normotensive subjects was, as suggested by deWardener and MacGregor, that the former would be in a “state of continuous correction of a slightly expanded extracellular volume,” resulting in a sustained elevation of NH (37). While this concept may have some general validity, the role of EDLF differs in various forms of hypertension (see below).

## Endogenous Digitalis-Like Factor

Because of the suggestion that NH might be an inhibitor of Na, K ATPase, it was subsequently referred to as “ouabain-like” or “digitalis-like.” This terminology became more than nomenclature as the field turned to proving the true digitalis-like nature of the circulating factor.

### The concept of endogenous drug-like compounds

The demonstration that the specificity and actions of some drugs were due to drug binding to stereospecific receptors had led to speculation that naturally occurring endogenous compounds existed that bound specifically to these receptors (38). The discovery of endogenous opioids was a direct result of this hypothesis (39). In 1976, Ginzler et al. proposed, as an extension of this concept, that antigen–antibody binding specificity might be analogous to drug-receptor binding specificity (40). That is, an antibody specific for a drug might recognize the same structure as the specific receptor for that drug, and could act as a “surrogate” receptor. This hypothesis had at least two interesting implications. First, antibodies to drugs might recognize endogenous compounds that utilize the same receptor as the drug; and second, antibodies to drugs (or endogenous compounds) might be used to block the effects of those compounds by displacing them from their receptor. The second possibility had already been anticipated by a number of investigators including the demonstration that digoxin antibodies would reverse the clinical manifestations of digoxin intoxication (41).

Based on these concepts, Gruber et al. showed in 1980 that plasma of volume expanded dogs but not euvolemic dogs contained a factor that cross reacted with digoxin antibodies in a specific fashion; i.e., the dose response curve in the digoxin radioimmunoassay (RIA) of the endogenous factor was parallel to that of authentic digoxin (42). Furthermore, plasma extracts containing the digoxin immunoreactive compound inhibited Na, K ATPase, providing further evidence for a true EDLF that had some structural and functional similarity to digoxin. The finding also suggested that digoxin RIAs could be used to study plasma levels of this factor and numerous studies of mammalian “digoxin-like” factor were soon published (43). However, studies using this approach are subject to non-specific cross reactivity of various interfering substances in the digoxin RIA and have led to some confusion (44, 45). Interestingly, the first demonstration of an endogenous substance that cross reacts with digoxin antibodies was in newborns, who were suspected of having been poisoned with digoxin (46).

### Characterization of EDLF

Subsequent to the work briefly described above, numerous attempts have been made to purify and identify the principal factor responsible for the digitalis-like factor demonstrated in volume expanded subjects. According to the initial hypothesis, a truly endogenous, natriuretic, hypertension promoting digitalis-like factor would have the following characteristics. First of all, it would be synthesized endogenously, and secreted under the control of relevant physiological or pathophysiological stimuli. Second, it would inhibit renal and vascular Na, K ATPase in a “ouabain-like” fashion; that is, it would bind to the same receptor and have similar effects on the enzyme as ouabain. Thirdly, its inhibition of renal tubular and vascular Na, K ATPase would cause natriuresis and vasoconstriction, respectively. Unfortunately, attempts to identify such a factor have been complicated by the fact that inhibition of the enzyme in various assay systems is a non-specific effect of many diverse compounds (47). As a result, numerous candidate structures have been identified, including steroids, lipids, peptides, and a variety of other novel compounds (45, 48, 49). A complete review of these reports is beyond the scope of this paper. Rather, we have chosen to focus on a class of compounds that are “digitalis-like” steroids, and which meet the theoretical criteria outlined above, with one exception. They have not been shown unequivocally to be “endogenous” since their synthetic pathway has not been completely elucidated, although preliminary studies suggest they are synthesized in the adrenal gland (50–53).

In 1991, Hamlyn et al. reported purification of a compound indistinguishable from ouabain by mass spectroscopy from 300 l of human plasma (54). Subsequent work seemed to confirm this observation and indicated that mammalian ouabain is present in multiple body fluids and tissues. However, the issue of whether mammalian tissues contain authentic ouabain has remained highly controversial (55–57) despite substantial evidence in support of this finding (58).

Amphibian species have been known for many years to synthesize a number of different steroids called bufodienolides that inhibit Na, K ATPase in a manner similar to the cardenolides (59). Dienolides differ from cardenolides in the structure of the lactone ring, which contains six members and two unsaturated double bonds compared to five members and one double bond in the cardenolides (43). Both cardenolides and dienolides have a 14 β hydroxyl group and a cis tertiary configuration of the C/D ring junction. Lichstein et al. identified a bufodienolide in toad skin and plasma as resibufogenin (60). They also demonstrated that the concentration of dienolides in toad skin was regulated by the salt content and osmolality of its aquatic environment (61, 62).

Bagrov et al. purified a digitalis-like compound from toad venom (63), which they subsequently identified as a previously described bufodienolide marinobufagenin (MBG) (64). Subsequently, purification of a substance from urine of patients after an acute myocardial infarction by high pressure liquid chromatography confirmed a structure indistinguishable from authentic MBG (65). Using a polyclonal antibody to toad MBG, they demonstrated increased concentration of a compound recognized by that antibody in plasma of volume expanded dogs (66) and rats (67), and patients with preeclampsia (68). Using antibodies specific for ouabain and MBG, they demonstrated that mammalian plasma contains both ouabain-like and MBG-like compounds (66). Subsequent work has demonstrated that the MBG-like compound meets essentially all the criteria originally postulated for the EDLF-type NH described above (69, 70).

Yoshika et al. have shown that MBG immunoreactivity secreted by adrenomedullary derived cells in tissue culture is composed of at least two compounds, MBG and a related compound marinobufotoxin (MBT) (71). MBT was shown to increase blood pressure when administered intraperitoneally to rats (71).

### EDLF and hypertension

Using multiple assays for EDLF, numerous studies have attempted to show some correlation between plasma EDLF levels and the blood pressure in human and experimental hypertension, details of which have been previously reviewed and are beyond the scope of this paper (69, 72–80) Many of these studies have relied on measurements using RIA technology with antibodies raised against the compound(s) of interest. As noted, these studies are subject to cross reactivity with compounds other than those to which the antibody was raised. Despite these problems, it seems likely that some EDLF(s) are elevated in some forms of human and experimental hypertension and may play a role in its pathophysiology.

Although most studies of the role of EDLF in hypertension have focused on the circulating factors, it seems likely that endogenous ouabain plays a role in certain types of hypertension through a pathway in the central nervous system (CNS). EDLF has been demonstrated in hypothalamic and pituitary extracts of rats, a compound (or compounds) that crossreacts with a polyclonal anti-ouabain antibody (81). Extensive studies by Huang et al. have shown increases in this compound in the hypothalamus of Dahl salt-sensitive rats (82), spontaneously hypertensive rats (SHR) (83), and normal rats in which blood pressure is increased by an increase in cerebrospinal fluid sodium concentration (84). The critical role of brain EDLF in each of these models was demonstrated by prevention of the rise in blood pressure by CNS administration of a commercially available antigen binding fragment (FAB) of an antidigoxin antibody known to cross react with EDLF (Digibind^®^) (see below). A further complexity in the hypertension promoting CNS EDLF system has been demonstrated by studies of central infusion of angiotensin II in rats. This hypertension provoking maneuver causes an increase in circulating endogenous ouabain through activation of a neuronal pathway involving central aldosterone (85).

An integrated role for both endogenous cardenolides and bufodienolides in hypertension in Dahl salt-sensitive rats is suggested by studies showing that release of MBG is controlled by the CNS ouabain pathway discussed above (86). Further studies on the role of CNS pathways in the pathophysiology of hypertension, and in controlling circulating endogenous digitalis-like factors (EDLFs) and blood pressure should be of interest.

### Reversal of EDLF effects by functional antagonists

Several functional antagonists of EDLF have been reported to reverse the effects of Na, K ATPase inhibition in various clinical and experimental situations, among which are anti-dogoxin and anti-ouabain antiserum, and two steroid compounds, rostafuroxin and resibufagenin, that may be receptor antagonists of one or another component of EDLF.

Digibind^®^ is a purified FAB of a sheep anti-digoxin antibody developed for the treatment of digoxin intoxication that is no longer available commercially. Studies using Digibind^®^ as a probe to assess the possible role of EDLF in hypertensive subjects assume that it will cross react with EDLF, and that in large enough doses will displace EDLF from its receptor, analogous to its effect in digoxin toxicity. A number of studies are compatible with this formulation. Krep et al. showed that Digibind^®^ reduced blood pressure in the DOCA-salt rat model (87). Kaide et al. obtained the same results in a 5/6 reduced renal mass model (88). In the latter study, no effect of Digibind^®^ on blood pressure was observed in sham-operated controls, suggesting that the blood pressure reduction was not due to some non-specific or toxic effect of Digibind^®^ such as an anaphylactoid reaction. Mann et al. had suggested the latter, but their studies were done with commercial preparations other than Digibind^®^ (89). In addition to these *in vivo* studies, Krep et al. showed that Digibind^®^ reversed the contraction response of isolated aorta to an EDLF isolated from peritoneal dialysis fluid (90). Digibind^®^ has also been reported to reduce blood pressure in several hypertension models when given directly into the CNS (84, 91), to block the natriuresis of saline infusion in dogs (66), and to improve neonatal outcomes in fetuses born to patients with preeclampsia (92).

Antibodies against other glycosides have also been shown to lower blood pressure in animal models. Anti-ouabain antibodies had no effect on blood pressure in normal rats (93). However, immunization against ouabain prevented the development of hypertension in Dahl salt-sensitive rats (94), and reduced sodium excretion in normal rats (93). Also, administration of MBG antibodies lowered blood pressure in Dahl salt-sensitive rats (95). These studies suggest that whatever EDLF might be, whether single or multiple compounds, it cross reacts with antibodies against several candidate EDLFs.

In addition to the work with antibodies, two possible receptor antagonists of EDLF have been reported. Rostafuroxin^®^ is a digitoxigenen derivative that selectively displaces ouabain from the Na, K ATPase receptor (96). The compound lowered blood pressure in Milan hypertensive rats (97), but failed to lower blood pressure in clinical trials in essential hypertension in humans (77). Resibufogenin (RBG) is a bufodienolide isolated from toad skin (60) and the traditional Chinese medication Chan Su made from dried toad venom (98). RBG has a structure that only differs from MBG by one oxygen atom on the 5-position of the steroid nucleus (99). Although RBG has “digitalis-like activity” (inhibits *in vitro* Na, K ATPase activity and ouabain binding) (60), RBG has been shown to lower blood pressure in rat models of preeclampsia and DOCA-salt hypertension (100), both of which have elevated levels of MBG. These data suggest RBG antagonizes at least some effects of MBG, but the exact mechanism has not been elucidated.

Although similar in structure, different cardenolides and bufodienolides have surprising and unpredictable species and tissue differences in their biological actions (101–103), including antagonism of each other’s effects (104–107). The mechanism of the latter phenomenon has been extensively studied by Song et al. (105). They proposed a complex set of models in which α and β subunits of Na, K ATPase can function as tetraprotomers with varying degrees of aggregation and pump inhibition. This concept may lead to an entirely new method of manipulating sodium pump function that could have clinical implications.

It should also be noted that ACTH induced hypertension in rats can be prevented by making the α-2 Na, K ATPase receptor for cardiac glycosides resistant to those compounds through genetic manipulation (108). This clearly implicates endogenous Na, K ATPase inhibitor(s) in the etiology of this type of experimental hypertension.

## Summary in Retrospect and Future Directions

The search for a factor that regulates renal sodium excretion in response to increased blood volume, a NH, stimulated by the experiments of deWardener et al. (4) has produced a huge body of literature, which can no longer be reviewed in a single article. This review, an update of an earlier one (109), emphasizes how the search for a NH converged with studies of the mechanism of increased vascular resistance in hypertension, resulting in the discovery of EDLF(s) (Figure 1). This important discovery has widespread physiologic and pathophysiologic implications and explains, at least in part, the highly conserved nature of the ouabain binding site on membrane Na, K ATPase.

The fact is that, after all the work briefly summarized here, an amazing degree of complexity to a relatively simple if naïve concept has emerged. Regarding the original NH proposal of deWardener et al., no single entity has emerged that fits their hypothesis, and new physiologically relevant natriuretic factors may yet be discovered (9). Atrial natriuretic peptides are clearly volume sensitive natriuretic factors that likely play some role in the renal response to acute volume expansion. ANPs have multiple effects including vasodilation, and one or more of these peptides probably play some role in the pathophysiology of hypertension and congestive heart failure (110). Interactions between ANP and several EDLFs have been demonstrated, which have potentially important physiologic and pathophysiologic implications (27, 111, 112), and further work in the area can be anticipated.

Ouabain, MBG, and other bufodienolides continue to be investigated as putative physiological regulators of renal and cardiovascular function, but no clear integrating hypothesis has yet emerged. MBG appears to fit the criteria for the circulating factor proposed by the original NH hypothesis better than ouabain (72), but ouabain is clearly involved in the regulation of sodium excretion by the CNS and further work on this system is anticipated (9, 73). The intrarenal mechanism by which bufodienolides cause natriuresis, which involves the recently discovered signaling function of Na, K ATPase, should be of ongoing interest (113). The synthetic pathways and tissue(s) origin for the various EDLFs have not yet been completely determined, and high priority should be given to this project (58). If EDLFs play a role in normal physiology and some hypertensive states, as current evidence indicates, interference with their synthesis or antagonism of their effects should provide further insights, and possibly new targets for antihypertensive drugs.

Anti-digoxin antibodies interact with a broad range of EDLFs (114) and have been utilized in both experimental animals and man, with some interesting results (87, 88, 115, 116). Although the commercial preparation used in most of these studies is no longer available, another commercially available preparation of digoxin antibodies (DigiFab^®^) has similar if not identical cross reactivity with EDLF (116–118). Finally, the ability of individual cardiotonic steroids (CTS) to interfere with the effects of other CTS has added another layer of complexity and suggests another novel approach to the study of and possible therapy of various conditions in which CTS may be involved (105).

Despite ongoing controversy regarding some of the details (58), the hypothesis that an endogenous regulator(s) of the ouabain binding site on the Na, K ATPase enzyme is involved in control of the cardiovascular system has proved to be immensely fertile. Further investigation of these endogenous regulators holds great promise for a better understanding of cardiovascular physiology, the pathophysiology of a diverse set of clinical disorders, including hypertension, preeclampsia, chronic renal failure, congestive heart failure, and cancer (70), and the intricate complexities of the ouabain binding site (108, 119, 120).

## Conflict of Interest Statement

The author declares that the research was conducted in the absence of any commercial or financial relationships that could be construed as a potential conflict of interest.
